# T cells are functionally not impaired in AML: increased PD-1 expression is only seen at time of relapse and correlates with a shift towards the memory T cell compartment

**DOI:** 10.1186/s13045-015-0189-2

**Published:** 2015-07-30

**Authors:** Frauke M. Schnorfeil, Felix S. Lichtenegger, Katharina Emmerig, Miriam Schlueter, Julia S. Neitz, Rika Draenert, Wolfgang Hiddemann, Marion Subklewe

**Affiliations:** Department of Internal Medicine III, Klinikum der Universität München, Munich, Germany; Clinical Cooperation Group Immunotherapy, Helmholtz Institute Munich, Munich, Germany; Division of Clinical Pharmacology, Department of Internal Medicine IV, Klinikum der Universität München, Munich, Germany; Division of Clinical Infectiology, Department of Internal Medicine IV, Klinikum der Universität München, Munich, Germany; Clinical Cooperation Group Pathogenesis of Acute Myeloid Leukemia, Helmholtz Institute Munich, Munich, Germany

**Keywords:** Acute myeloid leukemia (AML), T cell exhaustion, PD-1, Immune checkpoint molecules, Cancer immunotherapy

## Abstract

**Background:**

T cell function is crucial for the success of several novel immunotherapeutic strategies for the treatment of acute myeloid leukemia (AML). However, changes in phenotype and function of T cells have been described in various hematologic malignancies, mimicking T cell exhaustion known from chronic viral infections. Detailed knowledge about phenotype and function of T cells in AML patients at different stages of the disease is indispensable for optimal development and application of immunotherapeutic strategies for this disease.

**Methods:**

We used flow cytometry-based assays to characterize T cell phenotype and function in peripheral blood and bone marrow of AML patients at diagnosis, at relapse after intensive chemotherapy, and at relapse after allogeneic stem cell transplantation (SCT). Surface expression of CD244, PD-1, CD160, and TIM-3 was determined, and proliferation and production of IFN-γ, TNF-α, and IL-2 were measured.

**Results:**

We detected similar expression of inhibitory molecules on T cells from patients at diagnosis and from age-matched healthy controls. At relapse after SCT, however, PD-1 expression was significantly increased compared to diagnosis, both on CD4^+^ and CD8^+^ T cells. This pattern was not associated with age and cytomegalovirus (CMV) status but with a shift towards effector memory cells in relapsed AML patients. Proliferation and cytokine production assays did not reveal functional defects in T cells of AML patients, neither at diagnosis nor at relapse.

**Conclusion:**

We thus conclude that T cell exhaustion does not play a major role in AML. Immunotherapeutic strategies targeting autologous T cells thus have particularly good prospects in the setting of AML.

**Electronic supplementary material:**

The online version of this article (doi:10.1186/s13045-015-0189-2) contains supplementary material, which is available to authorized users.

## Background

Novel immunotherapeutic strategies are increasingly evolving for the treatment of acute myeloid leukemia (AML) [1, 2]. Many of these strategies inherently rely on the efficiency and functionality of autologous T cells; a detailed understanding of T cell function at different phases of the disease, e.g., at diagnosis and at relapse, is therefore of highest importance for their optimal application. These treatment options include multispecific antibody constructs such as CD33/CD3-bispecific T cell engaging (BiTE) antibodies [3–5] or other bispecific antibodies [6] that bring the CD3^+^ T cells in close contact with leukemic cells. Chimeric antigen receptors (CARs) or transgenic T cell receptors are introduced into patients’ T cells [7, 8]. And finally, immune checkpoint inhibitors such as PD-1/PD-L1-blocking antibodies unleash spontaneously pre-existing tumor- or leukemia-specific T cells [9–11].

However, different degrees of T cell dysfunctionality have been described in various hematologic malignancies, including adult T cell leukemia/lymphoma [12, 13], chronic myeloid leukemia [14, 15], and chronic lymphoid leukemia (CLL) [16–18]. These observations have recently been put into the context of T cell exhaustion, a state of T cell dysfunction that is defined by increased expression of several inhibitory receptors (CD244, PD-1, CD160, TIM-3, LAG-3) in combination with poor effector function (hypoproliferation, diminished cytokine production, impaired cytotoxicity) and finally apoptosis [19]. It was first described for antigen-specific T cells in chronic viral infection in mice [20–22] but has since been demonstrated in several human chronic infections, among others in patients with human immunodeficiency virus (HIV) [23–25]. Most of the data has been gathered on CD8^+^ T cells, but loss of effector function has also been described in virus-specific CD4^+^ T cells [26, 27].

CD244, PD-1, CD160, and TIM-3 are expressed on T cells and interact with their ligands on antigen-presenting cells upon TCR ligation, resulting in modulation of the T cell response. CD244, also known as 2B4, is a dual-function receptor that mediates activating or inhibitory signals depending on its expression level, extent of ligation, and relative amounts of certain adaptor molecules [28]. PD-1 limits T cell responses in infection [29] and autoimmunity [30]. Tumors can exploit this axis to escape the immune system by constitutive or inducible expression of PD-1 ligand [31]. CD160 coinhibits CD4^+^ T cells upon ligand binding [32], and when coexpressed with PD-1, it also inhibits CD8^+^ T cells [33]. TIM-3 inhibits Th1 T cells by binding to galectin-9 [34] and has been shown to promote T cell exhaustion during chronic viral infection [35] and in cancer [36, 37].

So far, data on T cell function or potential T cell exhaustion in AML is mainly based on the analysis of murine models. In a syngeneic AML model, it was reported that coexpression of PD-1 and TIM-3 defined a subset of CD8^+^ T cells deficient in cytokine production. During AML progression, the number of these cells increased [38].

In our study, we set out to analyze the phenotype (with a particular focus on the inhibitory molecules CD244, PD-1, CD160, and TIM-3) and function (proliferation, cytokine production) of peripheral blood (PB) and bone marrow (BM) T cells in AML patients at different stages of the disease (diagnosis, relapse after intensive chemotherapy, relapse after allogeneic stem cell transplantation (allo-SCT)) in comparison to healthy controls (HC) and untreated HIV-infected patients.

## Results

### Increased levels of CD244, PD-1, CD160, and TIM-3 were expressed on peripheral blood CD8^+^ and CD4^+^ T cells of untreated HIV patients compared to healthy controls

**Fig. 1:**
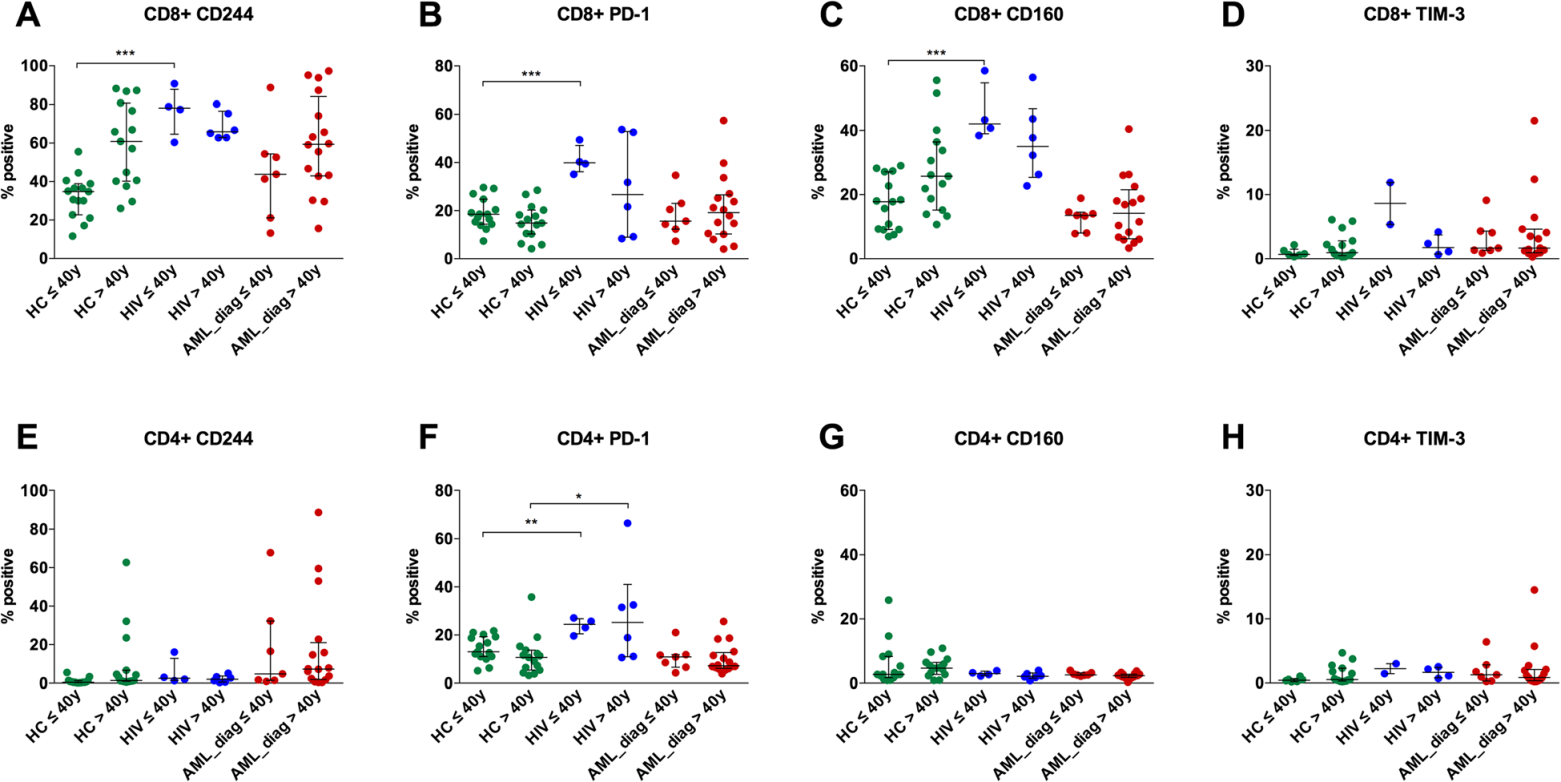
Similar expression of inhibitory molecules on peripheral blood T cells of AML patients at diagnosis compared to healthy controls. Expression of CD244 (**a**, **e**), PD-1 (**b**, **f**), CD160 (**c**, **g**), and TIM-3 (**d**, **h**) was measured on peripheral blood CD8^+^ (**a**–**d**) and CD4^+^ (**e**–**h**) T cells of 23 AML patients at diagnosis (*AML_diag*) in comparison to 30 healthy controls (*HC*) and 10 HIV patients (*HIV*), and percentages of positive cells were depicted. Samples were categorized according to age (≤40 vs. >40 years) within each group. Statistical differences were calculated to HC of the respective age cohort. *p ≤ 0.05; **p ≤ 0.01; ***p ≤ 0.001

CD244, PD-1, CD160, and TIM-3 are the most prominent inhibitory molecules upregulated in the context of T cell exhaustion. As a positive control, we analyzed 10 HIV patients before the start of highly active antiretroviral therapy (HAART) in comparison to 30 HC. In order to prevent an age bias, samples were categorized according to age (≤40 vs. >40 years) within both groups (Fig. 1).

CD8^+^ T cells of younger (≤40 years) HIV patients showed a strong increase in expression levels compared to the respective HC for CD244 (median of 78.2 vs. 34.8; *n* = 4 and *n* = 15; *p* = 0.0005; Fig. 1a), PD-1 (median of 39.9 vs. 18.5; *n* = 4 and *n* = 15; *p* = 0.0005; Fig. 1b), and CD160 (median of 42.0 vs. 17.8; *n* = 4 and *n* = 15; *p* = 0.0005; Fig. 1c). For TIM-3, there was only a non-significant trend towards higher expression (median of 8.6 vs. 0.7; *n* = 2 and *n* = 6; *p* = 0.07; Fig. 1d). In the older age group (>40 years), differences to HC were less pronounced and not statistically significant, but expression was still higher in the HIV patients for each molecule: CD244 (median of 65.9 vs. 60.9; *n* = 6 and *n* = 15; *p* = 0.52; Fig. 1a), PD-1 (median of 26.7 vs. 14.8; *n* = 6 and *n* = 15; *p* = 0.16; Fig. 1b), CD160 (median of 35.0 vs. 25.7; *n* = 6 and *n* = 15; *p* = 0.13; Fig. 1c), and TIM-3 (median of 1.7 vs. 0.9; *n* = 4 and *n* = 15; *p* = 0.53, Fig. 1d).

The expression of the inhibitory molecules on CD4^+^ cells was generally lower and more evenly distributed between both groups. An increased expression was found for PD-1 in HIV patients compared to HC, both in the younger (median of 24.4 vs. 13.0; *n* = 4 and *n* = 15; *p* = 0.004; Fig. 1f) and in the older cohort (median of 25.2 vs. 10.7; *n* = 6 and *n* = 15; *p* = 0.04; Fig. 1f). No relevant differences between both groups were found for CD244 (Fig. 1e), CD160 (Fig. 1g), and TIM-3 (Fig. 1h).

### Similar levels of CD244, PD-1, CD160, and TIM-3 were expressed on peripheral blood CD8^+^ and CD4^+^ T cells of AML patients at diagnosis compared to healthy controls

In order to evaluate the T cell status of AML patients, we started by immunophenotyping PB CD3^+^ T cells from 23 AML patients at diagnosis (AML_diag) in comparison to the 30 HC and 10 HIV patients mentioned above. Again, samples were categorized according to age (≤40 vs. >40 years) within each group (Fig. 1).

In contrast to HIV samples, no relevant increase in inhibitory molecule expression level on CD8^+^ T cells was found for the AML_diag samples compared to HC, neither for the younger cohort (CD244 median of 43.8; PD-1 median of 15.6; CD160 median of 13.5; TIM-3 median of 1.7; Fig. 1a–d) nor for the older cohort (CD244 median of 59.4; PD-1 median of 19.2; CD160 median of 14.2; TIM-3 median of 1.7; Fig. 1a–d). Similarly, expression levels of AML_diag on CD4^+^ cells were not elevated for any of the four molecules compared to HC (Fig. 1e–h).

### Increased levels of PD-1 were expressed on peripheral blood CD8^+^ and CD4^+^ T cells of AML patients at relapse after allogeneic SCT compared to diagnosis

**Fig. 2:**
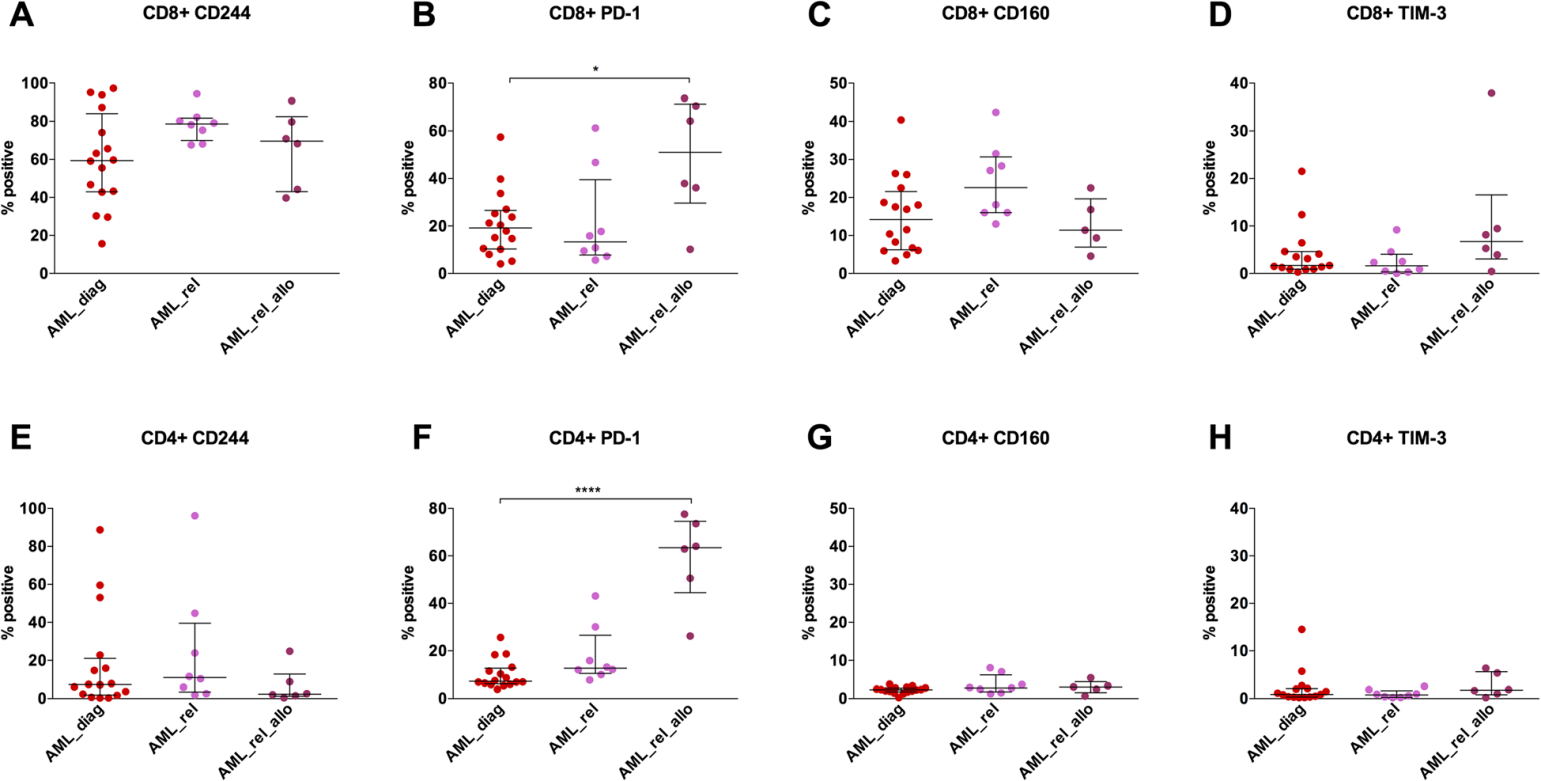
Increased PD-1 expression on peripheral blood T cells of AML patients at relapse after allogeneic SCT compared to diagnosis. Expression of CD244 (**a**, **e**), PD-1 (**b**, **f**), CD160 (**c**, **g**), and TIM-3 (**d**, **j**) was measured on peripheral blood CD8^+^ (**a**–**d**) and CD4^+^ (**e**–**h**) T cells of 8 patients with an AML relapse after intensive chemotherapy (*AML_rel*) and 6 patients with an AML relapse after allogeneic SCT (*AML_rel_allo*) in comparison to 16 AML patients at diagnosis (*AML_diag*), and percentages of positive cells were depicted. Patient selection was limited to the age cohort >40 years. Statistical differences were calculated to AML_diag. *p ≤ 0.05; ****p ≤ 0.0001

As inhibitory molecule expression in AML_diag was unaltered, we went on to study the relapse situation. Immunophenotyping of PB CD3^+^ T cells was performed for 8 patients with an AML relapse after intensive chemotherapy (AML_rel) and 6 patients with an AML relapse after allo-SCT (AML_rel_allo), each before the start of any cytotoxic treatment in the relapse situation, and results were compared to the 16 AML_diag specified above. Patient selection was limited to the age cohort >40 years (Fig. 2).

Both for CD8^+^ T cells and for CD4^+^ T cells, a strong upregulation of PD-1 was seen in AML_rel_allo compared to AML_diag (CD8^+^: median of 51.1 vs. 19.2; *p* = 0.02; Fig. 2b; CD4^+^: median of 63.5 vs. 7.2; *p* < 0.0001; Fig. 2f). The samples from AML_rel did not show a PD-1 upregulation (median of 13.3 and 12.7, respectively; Fig. 2b, f). For CD244, there was a trend towards higher expression in AML_rel (CD8^+^: median of 78.7 vs. 59.4; *p* = 0.05; Fig. 2a; CD4^+^: median of 11.0 vs. 7.3; *p* = 0.45; Fig. 2e), but no difference in AML_rel_allo (CD8^+^: median of 69.6; Fig. 2a; CD4^+^: median of 2.4; Fig. 2e). For CD160 (Fig. 2c, g) and TIM-3 (Fig. 2d, h), no significant differences in expression levels between the patient groups were found.

### Increased levels of PD-1 were expressed on bone marrow CD8^+^ and CD4^+^ T cells of AML patients at relapse after allogeneic SCT compared to diagnosis

**Fig. 3:**
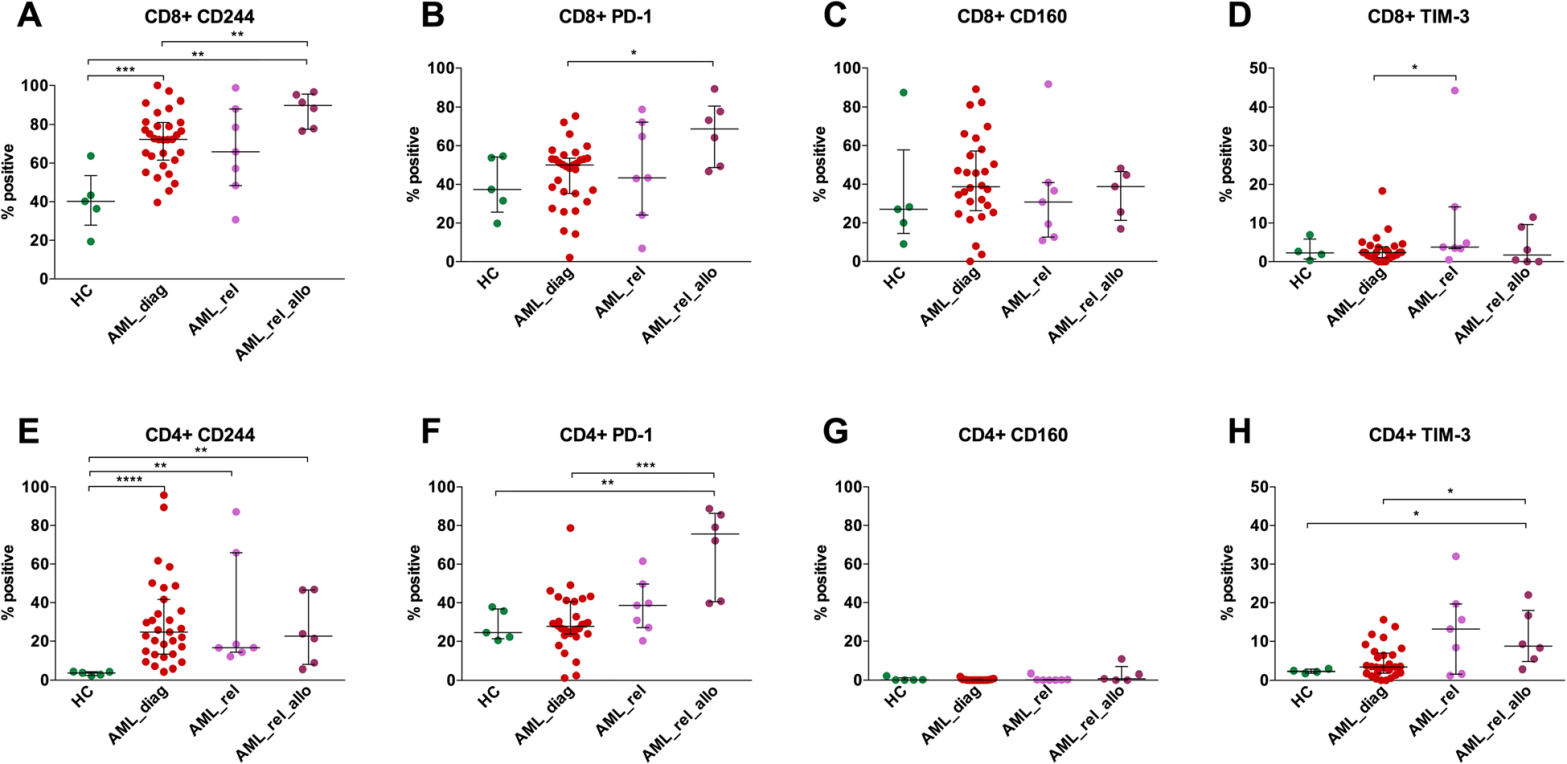
Upregulation of PD-1 expression on bone marrow T cells of AML patients at relapse after allogeneic SCT compared to diagnosis. Expression of CD244 (**a**, **e**), PD-1 (**b**, **f**), CD160 (**c**, **g**), and TIM-3 (**d**, **h**) was measured on bone marrow CD8^+^ (**a**–**d**) and CD4^+^ (**e**–**h**) T cells of 31 AML patients at diagnosis (*AML_diag*), 7 patients with an AML relapse after intensive chemotherapy (*AML_rel*), and 6 patients with an AML relapse after allogeneic SCT (*AML_rel_allo*) in comparison to 5 healthy controls (*HC*), and percentages of positive cells were depicted. Patient selection was limited to the age cohort >40 years. Statistical differences were calculated to HC and AML_diag. *p ≤ 0.05; **p ≤ 0.01; ***p ≤ 0.001; ****p ≤ 0.0001

As the microenvironment in the BM might influence T cell activation and exhaustion, we analyzed BM CD3^+^ T cells for 5 HC, 31 AML_diag, 7 AML_rel, and 6 AML_rel_allo. Analysis was limited to the age cohort >40 years (Fig. 3).

In contrast to PB CD3^+^ T cells, increased CD244 expression was clearly detectable in all AML BM samples, both on CD8^+^ and on CD4^+^ T cells. Expression on HC BM T cells was in the range of the PB samples (CD8^+^: median of 40.2; CD4^+^: median of 3.7), but expression in AML_diag (CD8^+^: median of 72.3; *p* = 0.0008; CD4^+^: median of 24.7; *p* < 0.0001), AML_rel (CD8^+^: median of 65.9; *p* = 0.07; CD4^+^: median of 16.6; *p* = 0.003), and AML_rel_allo (CD8^+^: median of 89.9; *p* = 0.004; CD4^+^: median of 22.6; *p* = 0.004) was upregulated (Fig. 3a, e). Only for CD8^+^ T cells, CD244 expression on AML_rel_allo was significantly higher than on AML_diag (*p* = 0.01; Fig. 3a).

Upregulation of PD-1 was found for AML_rel_allo in comparison to AML_diag, for CD8^+^ T cells (median of 68.7 vs. 50.0; *p* = 0.02; Fig. 3b) as well as for CD4^+^ T cells (median of 75.6 vs. 27.8; *p* = 0.0005; Fig. 3f). For CD4^+^ T cells, the difference of PD-1 expression in AML_rel_allo was also significant compared to HC (median of 24.5; *p* = 0.004; Fig. 3f), while the level of significance was not reached for CD8^+^ T cells in spite of a strong trend (HC: median of 37.4; *p* = 0.05; Fig. 3b).

CD4^+^ T cells from AML_rel_allo also showed increased expression of TIM-3 compared to HC (median of 8.8 vs. 2.3; *p* = 0.02) as well as compared to AML_diag (median of 3.3; *p* = 0.03; Fig. 3h), with a similar trend for AML_rel (median of 13.2 vs. 2.3 for HC; *p* = 0.30; median of 3.3 for AML_diag; *p* = 0.06; Fig. 3h). For CD8^+^ T cells, there was a significantly higher expression of TIM-3 in AML_rel compared to AML_diag (median of 3.8 vs. 2.4; *p* = 0.05; Fig. 3d). For CD160 (Fig. 3c, g), no significant differences in expression levels between HC and the various AML patient groups were found.

### CMV serostatus was not associated with expression pattern of inhibitory molecules

In order to exclude that the expression of CD244 and PD-1 on T cells is merely related to CMV status, we analyzed the expression of the inhibitory molecules on CD8^+^ T cells and on CD4^+^ T cells in 24 HC with confirmed seropositivity or seronegativity for CMV within two different age cohorts (≤40 vs. >40 years). No difference in expression levels with respect to CMV serostatus was found for CD244, PD-1, CD160, and TIM-3 (Additional file 1: Figure S1).

### Relapsed AML patients showed shift towards differentiated effector T cell phenotype

**Fig. 4:**
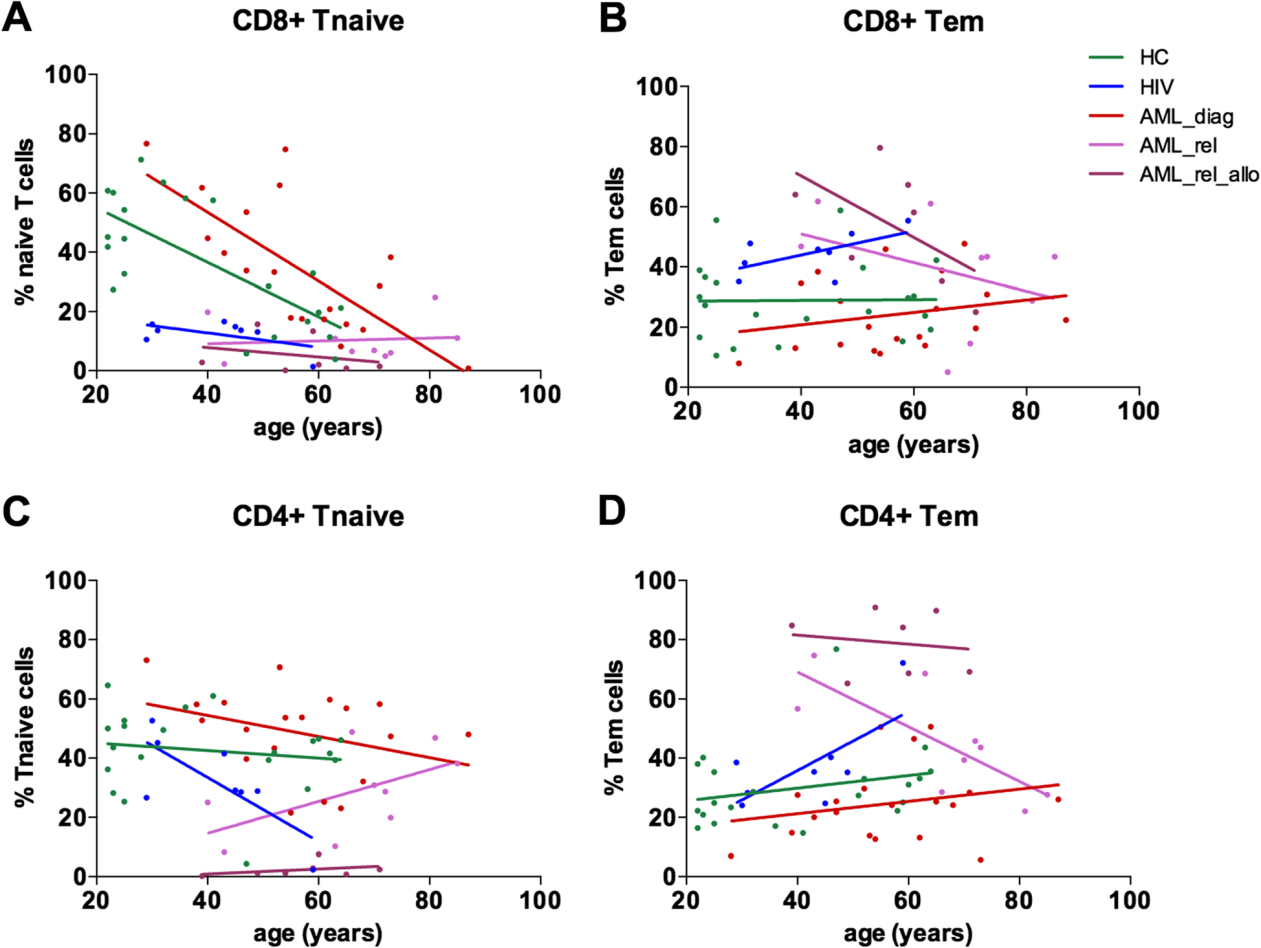
Skewing of T cells towards differentiated effector subtype in AML patients at relapse. Expression of CD45RA and CCR7 was measured on peripheral blood CD8^+^ (**a**, **b**) and CD4^+^ (**c**, **d**) T cells of 19 AML patients at diagnosis (*AML_diag*), 9 patients with an AML relapse after intensive chemotherapy (*AML_rel*), and 7 patients with an AML relapse after allogeneic SCT (*AML_rel_allo*) in comparison to 21 healthy controls (*HC*) and 8 HIV patients (*HIV*), and percentages of naïve (Tnaive; CD45RA^+^/CCR7^+^) and effector memory (Tem; CD45RA^−^/CCR7^−^) CD8^+^ and CD4^+^ T cells were plotted as a variable of age. Linear regression between T cell subset percentages and age was calculated and depicted

Next, we analyzed whether the distribution of T memory subsets and activation status differed between the various patient cohorts, as a skewing of the T cell compartment towards antigen-experienced memory cells might contribute to the observed increase in PD-1 expression in AML_rel_allo. CD45RA and CCR7 expression was measured on PB CD8^+^ and CD4^+^ T cells of 19 AML_diag, 9 AML_rel, and 7 AML_rel_allo, in comparison to 21 HC and 8 HIV patients, and percentages of naïve (Tnaive; CD45RA^+^/CCR7^+^) and effector memory (Tem; CD45RA^−^/CCR7^−^) CD8^+^ and CD4^+^ T cells were plotted as a variable of age (Fig. 4). For CD8^+^ T cells of HC, we observed a strong decline of Tnaive with age, a pattern that has been described previously [39] and that we could also confirm for AML_diag (Fig. 4a). The percentage of Tem was independent of age (Fig. 4b). In contrast, HIV patients as well as relapsed AML patients (both AML_rel and AML_rel_allo) showed lower percentages of Tnaive and higher percentages of Tem across all age groups (Fig. 4a, b). For CD4^+^ T cells, we similarly observed decreased Tnaive frequencies (Fig. 4c) and an increased proportion of Tem (Fig. 4d) for HIV patients and relapsed AML patients.

Additionally, the proportion of CD27^−^ cells was determined for CD8^+^ and CD4^+^ T cells of 19 AML_diag, 9 AML_rel, and 7 AML_rel_allo in comparison to 27 HC and 8 HIV patients to account for the more differentiated cells (Additional file 2: Figure S2). AML_diag showed a similar proportion of CD27^−^ cells as HC (CD8^+^: median of 19.8 vs. 14.7; *p* = 0.46; CD4^+^: median of 6.9 vs. 7.4; *p* = 0.92), while increased proportions were observed for HIV patients (CD8^+^: median of 46.8; *p* = 0.01; CD4^+^: median of 18.5; *p* = 0.006), AML_rel (CD8^+^: median of 39.7; *p* = 0.03; CD4^+^: median of 12.7; *p* = 0.04), and particularly AML_rel_allo (CD8^+^: median of 62.0; *p* = 0.03; CD4^+^: median of 30.6; *p* = 0.002).

### Differentiated effector T cells exhibited higher expression of CD244 and PD-1

**Fig. 5:**
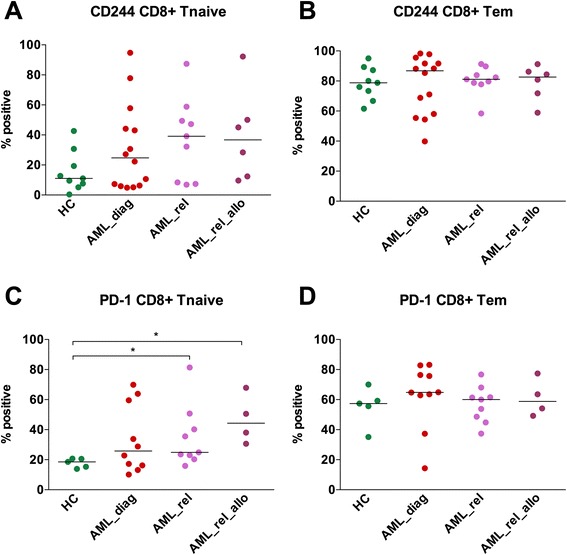
Elevated CD244 and PD-1 expression on differentiated effector T cells. Peripheral blood CD8^+^ T cells were classified into naïve (Tnaive; CD45RA^+^/CCR7^+^, (**a**, **c)**) and effector memory (Tem; CD45RA^−^/CCR7^−^, (**b**, **d**)) T cells, and expression of CD244 (**a**, **b**) and PD-1 (**c**, **d**) was measured for AML patients at diagnosis (*AML_diag*; *n* = 14 and *n* = 10), patients with an AML relapse after intensive chemotherapy (*AML_rel*; *n* = 9 and *n* = 9), and patients with an AML relapse after allogeneic SCT (*AML_rel_allo*; *n* = 6 and *n* = 4) in comparison to healthy controls (*HC*, *n* = 9 and *n* = 5). Percentages of positive cells were depicted. Statistical differences were calculated to HC. *p ≤ 0.05

In order to evaluate whether the differences in inhibitory molecule expression described above are due to the observed shift towards differentiated effector cells, we analyzed the expression of CD244 and PD-1 in the different CD8^+^ T cell subsets of AML_diag (*n* = 14 and *n* = 10), AML_rel (*n* = 9 and *n* = 9), and AML_rel_allo (*n* = 6 and *n* = 4; Fig. 5) in comparison to HC (*n* = 9 and *n* = 5). In general, similar levels of CD244 as well as PD-1 expression were observed within the same T memory subset of the different cohorts, with only minor increases of PD-1 expression in AML_rel (median of 24.9 vs. 18.5; *p* = 0.02) and AML_rel_allo (median of 44.3 vs. 18.5; *p* = 0.02) for Tnaive (Fig. 5c). Most importantly, the subset with the highest expression of the inhibitory molecules, Tem, showed similar expression levels in all cohorts (Fig. 5b, d).

### T cells of AML patients were functionally intact

**Fig. 6:**
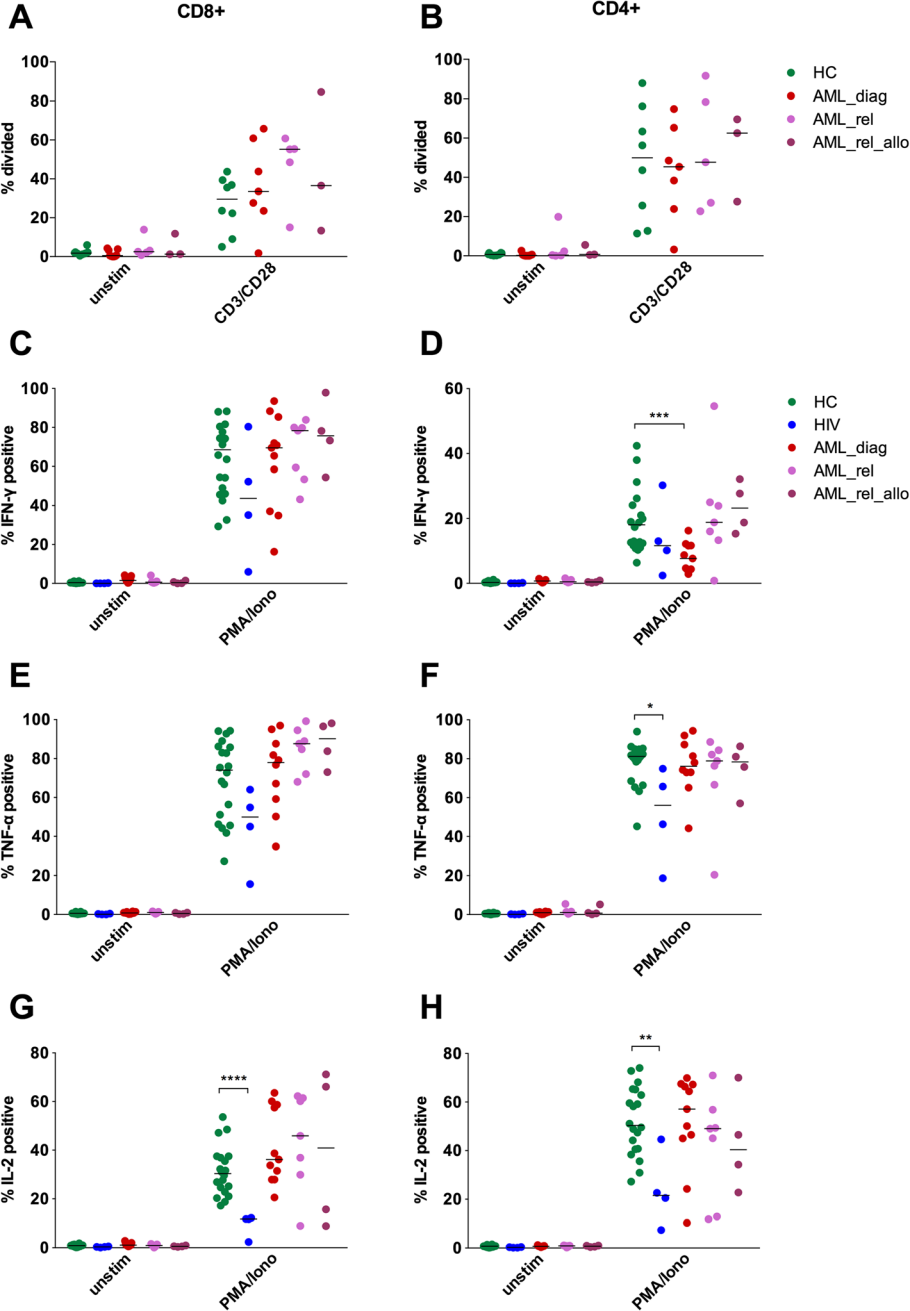
Functional integrity of T cells from AML patients. Proliferation (**a**, **b**) and production of IFN-γ (**c**, **d**), TNF-α (**e**, **f**), and IL-2 (**g**, **h**) with or without an unspecific stimulus were analyzed for peripheral blood CD8^+^ (**a**, **c**, **e**, **g**) and CD4^+^ (**b**, **d**, **f**, **h**) T cells of AML patients at diagnosis (*AML_diag*; *n* = 7 for proliferation and *n* = 11 for cytokines), patients with an AML relapse after intensive chemotherapy (*AML_rel*; *n* = 5 and *n* = 7), and patients with an AML relapse after allogeneic SCT (*AML_rel_allo*; *n* = 3 and *n* = 4) in comparison to healthy controls (*HC*; *n* = 8 and *n* = 20). Four HIV patients (*HIV*) served as an additional control for diminished cytokine production. Percentages of divided (**a**, **b**) or of positive (**c**–**h**) cells were depicted. Statistical differences were calculated to HC. *p ≤ 0.05; **p ≤ 0.01; ***p ≤ 0.001; ****p ≤ 0.0001

Finally, proliferation and cytokine production capacity of PB CD3^+^ T cells were tested for AML_diag (*n* = 7 and *n* = 11), AML_rel (*n* = 5 and *n* = 7), and AML_rel_allo (*n* = 3 and *n* = 4), with HC (*n* = 8 and *n* = 20) as control (Fig. 6). Additionally, 4 HIV patients served as a positive control in the cytokine production assay. No proliferation defect was found for any of the AML patient groups, neither for CD8^+^ (Fig. 6a) nor for CD4^+^ (Fig. 6b) T cells when stimulated with CD3/CD28 beads. Cytokine secretion of HIV T cells was impaired for IFN-γ (CD8^+^: median of 43.7 vs. 68.5; *p* = 0.22; Fig. 6c; CD4^+^: median of 11.6 vs. 18.1; *p* = 0.28; Fig. 6d), TNF-α (CD8^+^: median of 50.0 vs. 74.1; *p* = 0.06; Fig. 6e; CD4^+^: median of 56.0 vs. 81.2; *p* = 0.01; Fig. 6f), and IL-2 (CD8^+^: median of 11.7 vs. 30.4; *p* < 0.0001; Fig. 6g; CD4^+^: median of 21.6 vs. 50.4; *p* = 0.007; Fig. 6h), although statistical significance was not always reached due to the sample size. In contrast, cytokine secretion of all three AML patient cohorts was not impaired when stimulated with phorbol myristate acetate (PMA) and ionomycin, both for CD8^+^ and for CD4^+^ T cells, with the sole exception of decreased IFN-γ production of CD4^+^ T cells in AML_diag (median of 7.7 vs. 18.1; *p* = 0.0002; Fig. 6d).

## Discussion

Many novel immunotherapeutic strategies in AML including bispecific antibodies, CAR T cells, checkpoint inhibitors, and vaccinations rely on T cell function. For the optimal application and timing of these therapies, it is therefore of utmost importance to have detailed knowledge about the characteristics of T cells during the disease. Phenotypic changes and functional defects associated with T cell exhaustion have been described in patients with solid cancers [40, 41] and hematologic malignancies [12–15]. The most detailed studies of T cell status have been conducted in patients with CLL. An increased expression of CD244, PD-1, and CD160 was described on T cells of untreated CLL patients, preferentially on the CD8^+^ effector cells, accompanied by defects in proliferation and cytotoxicity, but at the same time, increased production of IFN-γ and TNF-α [17]. Within CLL patients in an early stage of disease, higher PD-1 positivity among CD8^+^ T cells was shown to be associated with worse prognosis [16]. Chemotherapy seemed to increase the expression of inhibitory receptors (CD244, PD-1) on T cells of CLL patients, while lenalidomide reversed this effect [18].

In comparison, little data is available on different aspects of T cell number, phenotype, and function in AML patients. An early study described an increase of some activation markers (HLA-DR, CD69, CD71, CD57) on T cells at diagnosis [42]. This was in line with data from gene expression profiling of T cells providing some hints at aberrant T cell activation in AML patients [43]. Patients in complete remission after intensive chemotherapy had normal CD8^+^ T cell counts but reduced numbers of CD4^+^ T cells and Tregs, and the proliferation of CD4^+^ T cells was not impaired [44]. During chemotherapy-induced leukopenia, however, when T cell counts are very low, the remaining T cells were shown to be functionally impaired and to need optimal costimulation in order to proliferate [45]. Antigen-specific CD8^+^ T cell responses are generally very rare in AML and were only studied after allo-SCT. In this setting, increased PD-1 levels on MiHA-specific CD8^+^ T cells [46] and the existence of a special subset of TNF-α^+^/IFN-γ^−^ T cells without further characterization [47] were described.

However, expression levels of inhibitory molecules associated with T cell exhaustion and the functional status of T cells at different phases of the disease have not been studied. We report here that CD8^+^ and CD4^+^ T cells in PB as well as in BM of AML patients at relapse after allo-SCT showed increased expression of PD-1, in contrast to T cells of AML patients at diagnosis.

Inhibitory molecules on CD4^+^ T cells have not been studied as broadly as on CD8^+^ T cells. However, the same molecules also seem to play a role in CD4^+^ T cells. Similarly to CD8^+^ T cells, persistent antigen exposure can induce a dysfunctional state in CD4^+^ T cells [48], which correlates with PD-1 expression [49, 50]. Tregs are unlikely to account for the observed increased expression of PD-1 on CD4^+^ T cells, as freshly isolated Tregs from healthy volunteers have been reported to express PD-1 solely intracellularly [51].

We were able to demonstrate that the inhibitory molecule expression pattern was independent of age and CMV status. Instead, high expression of CD244 and PD-1 was associated with T cell memory subset distribution. As had been described before [39], we found that the numbers of naïve T cells decline with age. However, we saw that patients with a relapse had a significantly higher proportion of effector memory T cells, independent of age. This shift was clearly detectable as an increase in the CCR7^−^/CD45RA^−^ subset as well as in CD27^−^ T cells, which have been described to be more differentiated [39, 52]. We could show that the subset of effector memory T cells inherently expressed higher levels of CD244 and PD-1, in healthy controls as well as across all patient cohorts. Therefore, we concluded that the increase in inhibitory molecule expression was most likely a surrogate for a shift towards differentiated effector T cells instead of a sign for T cell exhaustion. This is in line with the emerging notion that T cells with the characteristics defining exhaustion might rather be chronically activated [53].

Supporting our results, it has recently been shown in a study of healthy controls that inhibitory receptor expression depends more dominantly on differentiation and activation than on exhaustion of CD8^+^ T cells [53]. The increased expression of PD-1 on T cells of CLL patients was also found to be accompanied by a shift towards effector memory cells [54]. Similarly, in a very detailed study on T cell status in CLL, it was described that inhibitory molecule expression correlated with a skewing of T cells towards effector differentiation, although this study did not analyze patients after allogeneic SCT [17]. Supposedly, chronic antigen stimulation accounts for this shift in subsets. Importantly, however, functional defects were described for the T cells in CLL, particularly concerning proliferation and cytotoxicity [17], although it was demonstrated, on the other side, that CMV-specific CD8^+^ T cell function was not impaired [55].

In our study, we did not detect relevant functional impairment with respect to proliferation or cytokine production in T cells from AML patients at diagnosis or at relapse. Cytokine production was measured after antigen-unspecific T cell stimulation based on PMA and ionomycin, which bypasses TCR signaling. Unfortunately, antileukemic T cell responses were too rare to be measured. Even CD3/CD28 stimulation only resulted in very small T cell responses in such a setting. After stimulation with PMA and ionomycin, however, we observed compromised cytokine production in HIV patients. We therefore conclude that we can at least detect a functional defect downstream of TCR signaling this way. Our data are perfectly in line with a recent publication showing that the expression of PD-1 and CD244 on CD8^+^ T cells marks differentiated cells that intrinsically produce more cytokines [53]. The sole deficit found was a significantly decreased IFN-γ production of CD4^+^ T cells in diagnosis patients, but not in patients with an AML relapse. To our knowledge, this has not been described before and could constitute an important observation with respect to the evolving immunotherapeutic options, as the reversal of this defect by Th1-polarizing therapies could be part of the therapeutic effects. More unspecifically, serum levels of cytokines and chemokines have been measured before, and different groups found either identical [56] or reduced levels [57] of IFN-γ for untreated AML patients compared to healthy controls. Of course, our data applies to bulk T cells; therefore, antigen-specific T cells occurring at low frequencies could potentially vary from this pattern.

## Conclusions

From the data presented here, we conclude that AML patients in the relapse situation show a skewing of their T cell compartment from naïve towards differentiated effector cells, which is accompanied by an upregulation in inhibitory molecule expression, but not by a defect in their functional capacity. T cells of AML patients at diagnosis, in contrast, are not significantly different from those of age-matched healthy controls, suggesting that increased T cell differentiation could be induced by a long-standing contact with AML cells in the sense of chronic stimulation. Therefore, while NK cell defects of AML patients have frequently been reported [58], our data implies that immunotherapeutic strategies relying on the functionality of autologous T cells, such as the application of bispecific antibodies or therapeutic vaccination, have particularly good prospects in the setting of AML.

## Methods

### Sample collection

After written informed consent in accordance with the Declaration of Helsinki and approval by the Institutional Review Board of the Ludwig-Maximilians-Universität (Munich, Germany), PB or BM samples were collected from HC and AML patients at diagnosis or relapse before the start of treatment. Patients were treated according to the German AML-CG treatment recommendations. Samples of HIV patients before start of highly active antiretroviral therapy (HAART) were collected at the Division of Clinical Infectiology at the Klinikum der Universität München. The cytomegalovirus (CMV) serostatus was determined at the Virology Department of the Max-von-Pettenkofer-Institute of the Ludwig-Maximilians-Universität.

### Immunophenotyping of lymphocytes

Mononuclear cells (MCs) were isolated from PB or BM by Ficoll density gradient centrifugation. Immunofluorescent staining of cell surface antigens was performed using the following fluorescence-conjugated monoclonal antibodies: CD244 (PE or APC, C1.7), PD-1 (APC or Brilliant Violet 421, EH12.7H7), CD3 (AlexaFluor 488, UCHT1), CD45RA (Brilliant Violet 421, HI100), CCR7 (PE, G043H7), CD27 (APC, O323; all BioLegend, San Diego, CA, USA), CD160 (APC, 688327), TIM-3 (PE, 344823; both R&D Systems, Minneapolis, MN, USA), CD8 (PerCP-eFluor 710, SK1; eBioscience, San Diego, CA, USA), and CD4 (APC-H7, RPA-T4; BD Biosciences, San Jose, CA, USA). Corresponding isotype controls were used. PB and BM cells were analyzed using LSR II (BD Biosciences, Heidelberg, Germany) and Navios (Beckman Coulter, Krefeld, Germany) instruments, respectively. Post-acquisition analysis was performed using FlowJo software (version 9.6; Tree Star, Ashland, OR, USA).

### CFSE proliferation assay

CD3^+^ T cells were isolated from fresh or frozen PBMCs of HC and AML patients by MACS (Miltenyi Biotec, Bergisch Gladbach, Germany) labeled with 0.625 μm CFSE (Life Technologies, Carlsbad, CA, USA), and cultured in the presence of Dynabeads Human T-Activator CD3/CD28 (Life Technologies GmbH, Darmstadt, Germany) at a bead-to-cell ratio of 1:3 for 7 days. Unstimulated cells served as negative control. Harvested cells were then stained with antibodies for CD3 (APC, UCHT1; BioLegend), CD4 (APC-H7, RPA-T4; BD Biosciences), and CD8 (PerCP-eFluor 710, SK1; eBioscience).

### Cytokine production assay

Isolated CD3^+^ T cells were stimulated with PMA (20 μg/ml) and Ionomycin (750 ng/ml; both Sigma-Aldrich, St. Louis, MO, USA) after overnight resting. After 1 h, Golgi stop solution consisting of Monensin at 25 μM and Brefeldin A at 10 μg/ml (both Sigma-Aldrich) was added for additional 5 h. Harvested cells were surface stained for CD3 (AlexaFluor 488, UCHT1; BioLegend), CD4 (APC-H7, RPA-T4; BD Biosciences), and CD8 (PerCP-eFluor 710, SK1; eBioscience) and intracellularly with the BD Cytofix/Cytoperm Kit (BD Biosciences) and antibodies for IFN-γ (PE, B27), IL-2 (Brilliant Violet 421, MQ1-17H12), and TNF-α (APC, MAb11; all BioLegend).

### Statistical analysis

Data was analyzed using Prism 6 (GraphPad Software, La Jolla, CA, USA) and is reported in scatter plots. Statistical significance of differences was determined using the Mann-Whitney *U* test. *p* ≤ 0.05 was considered statistically significant (* in all figures), *p* ≤ 0.01 is designated with **, *p* ≤ 0.001 with ***, and *p* ≤ 0.0001 with ****.

## Additional files

Additional file 1: Figure S1. No association of inhibitory molecule expression with CMV serostatus. Expression of CD244 (A, E), PD-1 (B, F), CD160 (C, G), and TIM-3 (D, H) was measured on peripheral blood CD8^+^ (A–D) and CD4^+^ (E–H) T cells of 24 healthy controls (*HC*), and percentages of positive cells were depicted. Samples were categorized according to age (≤40 vs. >40 years) and CMV serostatus (CMV^+^ or CMV^−^). No statistical differences between CMV^+^ and CMV^−^ were found. (TIFF 1310 kb) Additional file 2: Figure S2. Increased percentages of differentiated CD27^−^ T cells in AML patients at relapse. Expression of CD27 was measured on peripheral blood CD8^+^ (A) and CD4^+^ (B) T cells of 19 AML patients at diagnosis (*AML_diag*), 9 patients with an AML relapse after intensive chemotherapy (*AML_rel*), and 7 patients with an AML relapse after allogeneic SCT (*AML_rel_allo*), in comparison to 27 healthy controls (*HC*) and 8 HIV patients (*HIV*), and percentages of CD27^−^ cells were depicted. Statistical differences were calculated to HC. **p* ≤ 0.05; ***p* ≤ 0.01. (TIFF 385 kb)

## Abbreviations

allo-SCT: allogeneic stem cell transplantation
AML: acute myeloid leukemia
AML_diag: AML patients at diagnosis
AML_rel: patients with an AML relapse after intensive chemotherapy
AML_rel_allo: patients with an AML relapse after allo-SCT
BM: bone marrow
CAR: chimeric antigen receptor
CLL: chronic lymphoid leukemia
CMV: cytomegalovirus
HAART: highly active antiretroviral therapy
HC: healthy control(s)
HIV: human immunodeficiency virus
MC: mononuclear cell
PB: peripheral blood
PMA: phorbol myristate acetate
