# Supplementary figures and images for: T cells are functionally not impaired in AML: increased PD-1 expression is only seen at time of relapse and correlates with a shift towards the memory T cell compartment
Source: J Hematol Oncol. 2015 Jul 30;8:93. doi: 10.1186/s13045-015-0189-2 (PMC4518596; doi:10.1186/s13045-015-0189-2)

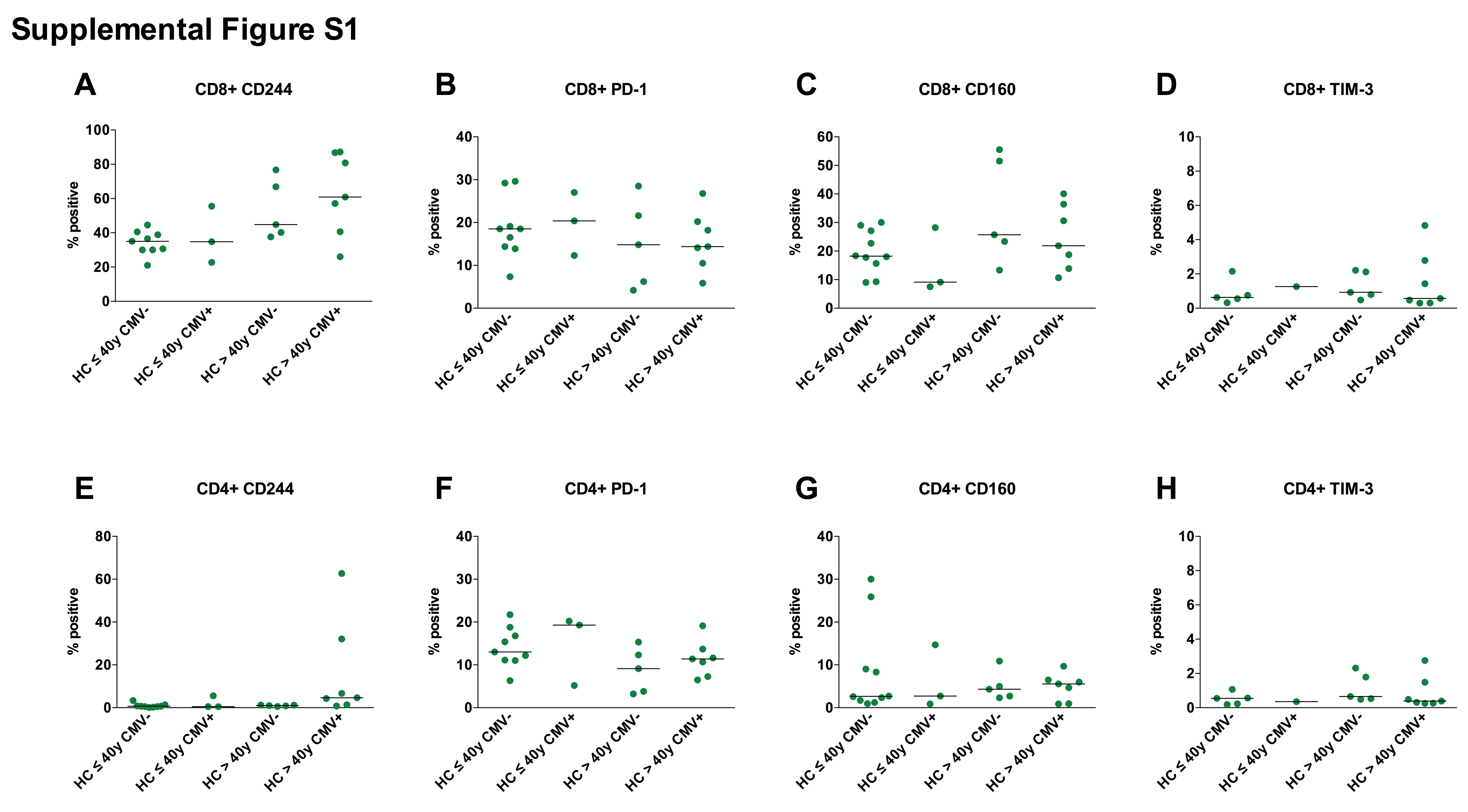

Supplement: Additional file 1: Figure S1. — No association of inhibitory molecule expression with CMV serostatus. Expression of CD244 (A, E), PD-1 (B, F), CD160 (C, G), and TIM-3 (D, H) was measured on peripheral blood CD8+ (A–D) and CD4+ (E–H) T cells of 24 healthy controls (HC), and percentages of positive cells were depicted. Samples were categorized according to age (≤40 vs. >40 years) and CMV serostatus (CMV+ or CMV−). No statistical differences between CMV+ and CMV− were found. (TIFF 1310 kb) [file 13045_2015_189_MOESM1_ESM.tiff]

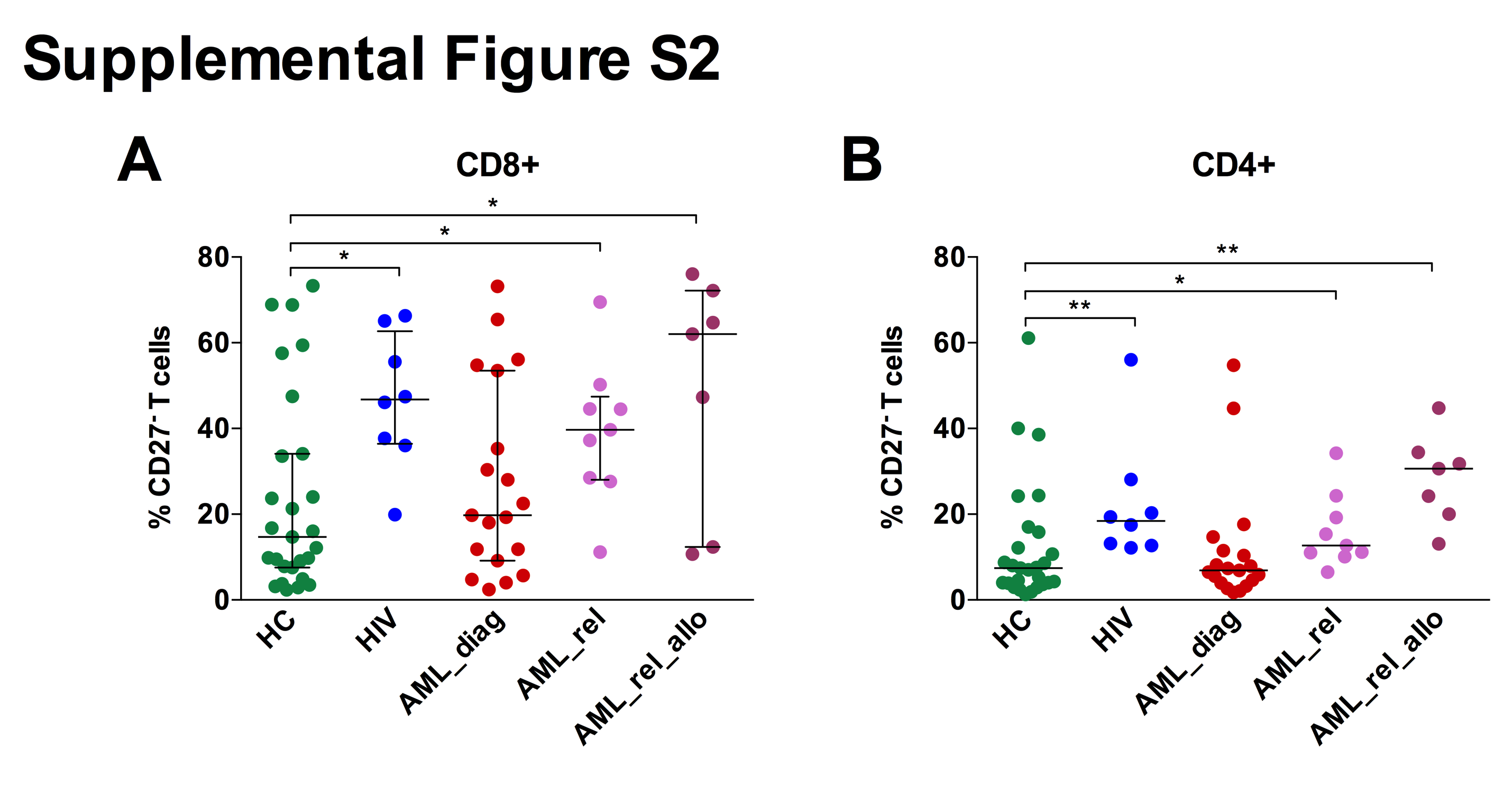

Supplement: Additional file 2: Figure S2. — Increased percentages of differentiated CD27− T cells in AML patients at relapse. Expression of CD27 was measured on peripheral blood CD8+ (A) and CD4+ (B) T cells of 19 AML patients at diagnosis (AML_diag), 9 patients with an AML relapse after intensive chemotherapy (AML_rel), and 7 patients with an AML relapse after allogeneic SCT (AML_rel_allo), in comparison to 27 healthy controls (HC) and 8 HIV patients (HIV), and percentages of CD27− cells were depicted. Statistical differences were calculated to HC. *p ≤ 0.05; **p ≤ 0.01. (TIFF 385 kb) [file 13045_2015_189_MOESM2_ESM.tiff]
